# A nanonewton force sensor using a U-shape tapered microfiber interferometer

**DOI:** 10.1126/sciadv.adk8357

**Published:** 2024-05-29

**Authors:** Ling Chen, Bin Liu, Christopher Markwell, Juan Liu, Xing-Dao He, Zabih Ghassemlooy, Hamdi Torun, Yong-Qing Fu, Jinhui Yuan, Qiang Liu, Gerald Farrell, Qiang Wu

**Affiliations:** ^1^ State Key Laboratory of Information Photonics and Optical Communications, Beijing University of Posts and Telecommunications, Beijing 100876, China.; ^2^ Key Laboratory of Opto-Electronic Information Science and Technology of Jiangxi Province, Nanchang Hangkong University, Nanchang 330063, China.; ^3^ Optical Communications Research Group, Faculty of Engineering and Environment, Northumbria University, Newcastle Upon Tyne NE1 8ST, UK.; ^4^ School of Control Engineering, Northeastern University at Qinhuangdao, Qinhuangdao 066004, China.; ^5^ School of Electrical and Electronic Engineering, City Campus, Technological University Dublin, Dublin D07 ADY7, Ireland.

## Abstract

Nanomechanical measurements, especially the detection of weak contact forces, play a vital role in many fields, such as material science, micromanipulation, and mechanobiology. However, it remains a challenging task to realize the measurement of ultraweak force levels as low as nanonewtons with a simple sensing configuration. In this work, an ultrasensitive all-fiber nanonewton force sensor structure based on a single-mode–tapered U-shape multimode–single-mode fiber probe is proposed and experimentally demonstrated with a limit of detection of ~5.4 nanonewtons. The use of the sensor is demonstrated by force measurement on a human hair sample to determine the spring constant of the hair. The results agree well with measurements using an atomic force microscope for the spring constant of the hair. Compared with other force sensors based on optical fiber in the literature, the proposed all-fiber force sensor provides a substantial advancement in the minimum detectable force possible, with the advantages of a simple configuration, ease of fabrication, and low cost.

## Teaser

The measurement of ultraweak force levels as low as nanonewtons is achieved using a U-shape tapered microfiber interferometer.

## INTRODUCTION

There is a strong demand for techniques and systems that can realize accurate, highly sensitive measurement and micromanipulation of small contact forces with applications in nanoscale science. In the biomolecular domain, mechanical cues initiate several cellular functions and can alter the behavior of cells at a molecular level in addition to biochemical effects. Mechanical signals are thus as important as chemical and electrical signals in biology. For example, molecular interactions including receptor-ligand interactions are regulated mechanically and have important implications in immunology and drug discovery such as in the design of drug-target complexes that stress bacterial cell membranes, which have important applications in neutralizing drug-resistant “superbugs” (*1*). In addition to molecular interactions, the mechanics of intracellular processes can be used to differentiate cancerous cells from normal cells (*2*), for example, Sarah *et al*. and Iyer *et al*. (*3*, *4*) measured and compared the mechanical responses of cancerous and normal cells with an atomic force microscope (AFM), in the study micro/nanoscale mechanics in tumor development and metastasis. Given the capability of AFM to interact with biological entities at a nanoscale level with a sub-nano level measurement capability, AFM is the most commonly used instrument to characterize the mechanical properties of soft materials in a wide range of mechanobiology applications (*5*–*9*). In particular, controlling the distance between a probing microscale cantilever and a sample, while measuring the interaction forces, allows for the quantitative analysis of contact events. The force-distance curves obtained from these experiments are characterized by two regions. First, the indentation region, where the cantilever is actuated toward the sample, can be used to extract information regarding sample elasticity, deformation, and dissipation. Second, the retraction region, where the cantilever is actuated away from the sample so as to break contact with the sample, can provide useful information about the adhesion dynamics and interaction forces between the stylus and sample (*10*, *11*). For example, Cortelli *et al*. (*12*) used an AFM to investigate the nanomechanics of thin gold layers deposited on a silicone elastomer substrate, and Mertens *et al*. (*13*) used the AFM for the nanomechanical detection of the interaction between *Escherichia coli* and bacteriophage T7. AFM-based imaging and force spectroscopy have underpinned many nanoscale research investigations due to the ability of AFM to provide high resolution and very high sensitivity, with the advantages of simple sample preparation and the ease with which information can be extracted from the surface morphology of the sample. However, conventional AFM instruments are usually physically large, expensive, and complex and require highly trained operators in laboratories. As a result, AFM-based technology has several disadvantages, especially when utilization outside of a laboratory environment is envisaged, where space is limited, or where multiple samples need to be analyzed simultaneously. Researchers have proposed several different technologies which can provide similar functionality to AFM, but which address the disadvantages of AFM. One of the earliest reports in 2006 by Iannuzzi *et al*. (*14*) proposed a fiber-top cantilever AFM and demonstrated that its performance was comparable to that of a commercially available AFM. Later, Gellineau *et al*. (*15*) designed an optical fiber AFM based on a photonic crystal fiber force sensor for in vivo imaging which had a resolution of 10 pN/Hz. Miyahara *et al*. (*16*) eliminated the problems associated with the spurious mechanical resonances induced by the piezoelectric transducer usually used in a frequency modulation AFM system by instead using an optical excitation force to excite the AFM cantilever, in which a fiber-optic interferometer system is used to detect cantilever deflection. Chang *et al*. (*17*) combined an all-optical miniaturized ferrule-tip cantilever probe with a high-speed AFM system to achieve high-performance imaging. In recent years, micro/nanoelectromechanical system (MEMS/NEMS) force sensors have been investigated for important roles in minimally invasive surgery, cell manipulation, biomedical science, and microscale drug delivery. The nanomechanical cantilevers, used within ultrasensitive MEMS/NEMS devices, have been developed specifically to provide miniaturization and real-time monitoring (*18*). However, MEMS/NEMS components operate electrically and are thus susceptible to electromagnetic interference.

There are also a variety of other technologies which have gradually been developed for contact force measurement. For example, Zhang *et al.* (*19*) used an optically controlled hydrodynamic manipulation method for measuring the weak force of a single microscopic particle. However, the accuracy of this technology is greatly affected by local electric and magnetic fields. Optical/magnetic traps are one of the main nanomechanical tools in which force can be exerted without a need for direct contact with specimens (*20*–*22*). However, despite the advantages of noncontact operation, irradiating the targets with focused light in optical traps can be detrimental for cells and biological molecules, for example, because the absorption of optical energy can lead to damage as a result of photothermal effects for targets and their surroundings (*23*). Magnetic tweezers have been extensively investigated but suffer from the disadvantages of magnetic field hysteresis and the generation of heat during operation (*24*). Lastly, a variety of other molecular force probes including mechanophores, quantum dots, fluorescent pairs, and molecular rotors have been proposed to measure intracellular stresses (*25*, *26*). However, each of these probes has associated disadvantages, for example, the operating times of fluorescence-based techniques are very short due to photo-instability, resulting in a substantial challenge when quantifying forces with high spatial and mechanical resolutions.

Compared with other force sensors, optical fiber force sensors have many advantages, such as high sensitivity, immunity to electromagnetic interference, good stability, small size, and good biocompatibility. As a result, a variety of optical fiber force sensors have been developed and applied in various areas. In 2018, Wu *et al.* (*27*) formed a single-mode fiber (SMF) into a balloon-like shape to act as a modal interferometer for force sensing, achieving a maximum force sensitivity of 24.9 pm/μN. In the same year, Shen *et al.* (*28*) used tilted fiber Bragg gratings for the measurement of millinewton-level surface tension forces. Later in 2020, Pevec *et al.* (*29*) proposed a miniature all-fiber Fabry-Perot (FP) sensor for the measurement of force, demonstrating a resolution of ~0.6 μN within a measurement range of ~0.6 mN. Recently, Zou *et al.* (*30*) reported a fiber-tip polymer clamped-beam probe, which showed a force sensitivity of 1.51 nm/μN and a detection limit of 54.9 nN over a measurement range of ~2.9 mN. However, these reported force measurement technologies do have some shortcomings. Most of them rely on expensive equipment such as a femtosecond laser for fabrication and since they operate by monitoring the shift of a dip wavelength caused by the applied force, their response time is relatively slow. These shortcomings greatly limit their practical applications.

An optical fiber interferometer based on a tapered single-mode–multimode–single-mode (SMS) fiber structure has been widely researched as a sensing structure due to its ultrahigh sensitivity and ease of fabrication (*31*). In this paper, a U-shape tapered SMS fiber structure is proposed which can act as a probe to successfully detect nanonewton-level force. Contact force measurement of human hair is used as a practical demonstration of the effectiveness of the approach, with a result that agrees well with that measured by an AFM. The U-shape tapered microfiber interferometer can detect contact force as low as ~5.4 nN, which is one of the lowest detection limits for an optical fiber-based force sensor reported so far which the authors are aware of. The very low detection limit and the ability to operate while immersed in a liquid raise the possibility that the proposed microfiber interferometer can be successfully used for nanomechanical measurements of biological cells, elastography of tissues, and applications in the area of high-precision material science.

## RESULTS

### Sensor calibration using AFM

**Fig. 1.**
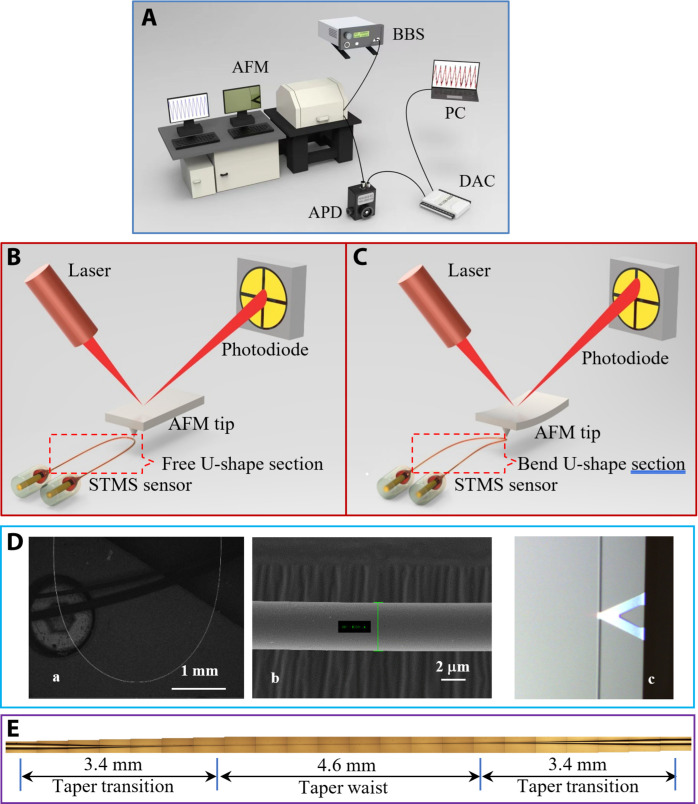
Schematic experimental setup. A schematic diagram of (**A**) experimental setup of the AFM calibration sensing system. BBS, broadband optical source; APD, amplified photodetector; DAC, data acquisition card; PC, personal computer. (**B**) Before contact between the AFM tip and fiber. (**C**) AFM tip slightly deforms after the probe contacts the U-shape fiber section. (**D**) SEM images of a typical U-shape tapered microfiber interferometer: (a) U-shape fiber section, (b) partially enlarged image of the U-shape fiber section (diameter, 4.222 μm), and (c) microscope image of the tip in contact with the U-shape tapered fiber. (**E**) An example of an optical microscope image of a fiber taper section of one of the U-shape tapered fibers used in the experiment.

Figure 1A shows a schematic diagram of the experimental setup for force calibration of the proposed sensor using AFM. From Fig. 1A, the light from a broadband optical source (S5FC1550P-A2, Thorlabs) propagates through the single-mode–tapered-multimode–single-mode (STMS) fiber structure and is then collected by an Indium gallium arsenide (InGaAs)–amplified photodetector (InGaAs-APD; Thorlabs: PDA10CS-EC, 700 to 1800 nm), which converts the optical signal to an electrical signal, connected to a data acquisition card (MC Measurement, USB-1608FS-Plus), which, in turn, is connected to a personal computer (PC). A commercial AFM system (Bruker Dimension Icon) was used in the experiments to provide a calibrated source of dynamic force. The AFM cantilevers were actuated against fiber samples by applying triangular signal waveforms with an amplitude of up to 12 μm and a frequency of 1 Hz. AFM cantilevers (Bruker, SCANASYST-AIR) were used during the experiments. The spring constant (*k*) of the cantilever was calibrated as 0.4 N/m using the thermal tuning method. The AFM tip attached to a piezo actuator was carefully aligned to remain in contact with the U-shape microfiber, while the AFM cantilever was displaced with a travel range of up to 12 μm using a periodic ramp signal. Before the cantilever tip was placed in contact with the U-shape section of the STMS fiber structure, the tapered optical fiber did not display any deformation, as shown in Fig. 1B. However, on contact with the cantilever tip, the tapered optical fiber deformed causing the change of the received optical signal level because the tip exerted a contact force on the U-shape fiber section, as shown in Fig. 1C. The larger the deformation of the U-shape fiber section, the larger the signal level variation. A scanning electron microscope (SEM) image of a typical U-shape tapered fiber section, which has a fiber waist diameter of 4.222 μm, is shown in Fig. 1D, showing both the overall structure (Fig. 1D-a) and enlarged view (Fig. 1D-b). The microscope image of the cantilever tip contacting the optical fiber is shown in Fig. 1D-c. Figure 1E shows a typical example of an optical microscope image of the fiber taper section used in the experiment.

### Mechanical sensing performance of the U-shape microfibers

In our experiments, three different U-shape STMS fiber structures with different taper waist diameters (see Table 1) were fabricated for test purposes.

**Table 1.** Parameters of the three U-shape STMS fiber sensors.

Sensor type	Taper waist diameter	Taper waist length	Transition length	Fiber sensor spring constant
Sensor 1	4.222 μm	4.6 mm	3.4 mm	4.00 × 10^−3^ N/m
Sensor 2	4.606 μm	4.4 mm	3.2 mm	5.25 × 10^−3^ N/m
Sensor 3	5.917 μm	4.3 mm	3.0 mm	11.83 × 10^−3^ N/m

**Fig. 2.**
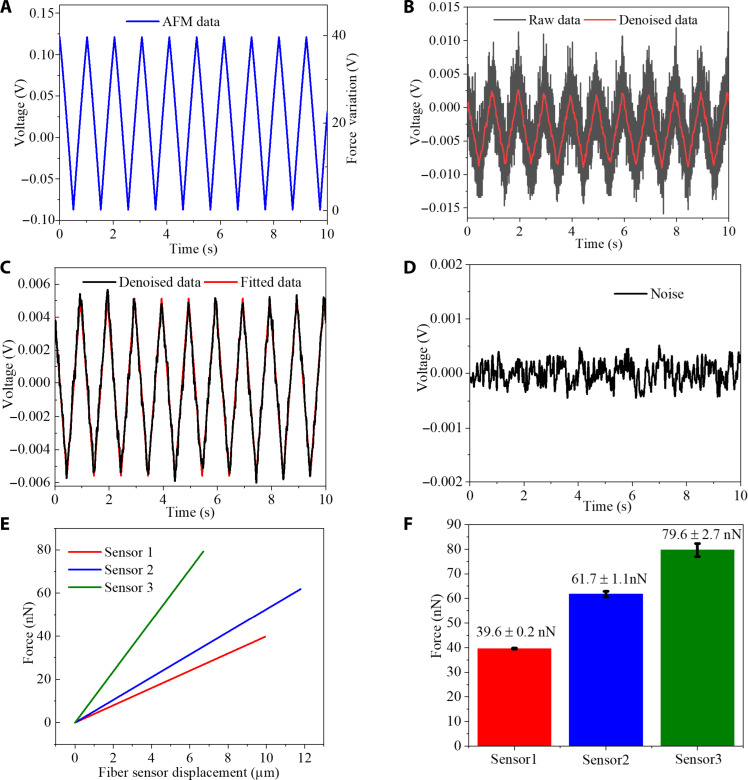
AFM calibration of the U-shape STMS fiber sensor. (**A**) Signal response of the AFM. (**B**) Signal response of U-shape STMS fiber structure with bend diameter of 3 mm when a periodic triangular movement waveform is applied to the U-shape STMS by the AFM tip driven by a piezo actuator and with noise-filtering of the data (low-pass filtering by removing frequencies >25 Hz). (**C**) The noise-filtered data and fitted signal data points from the U-shape STMS fiber structure with a bend diameter of 3 mm corresponding to (B). (**D**) The noise only of the experimental system. (**E**) The relationship between the deflection distance of the fiber and tip and the force exerted on the three fiber sensors. (**F**) Calibration repeatability test results for the three fiber sensors.

Figure 2 (A to D) shows a typical example of the measured signal responses from the AFM and from one of the U-shape STMS fiber structures (sensor 1 in Table 1) with a bend diameter of 3 mm, in which a periodic triangular movement waveform (10-μm peak-peak in amplitude at a frequency of 1 Hz) is applied to the U-shape STMS by a piezo actuator in the AFM. The associated video material is presented in the Supplementary Materials.

As the displacement of the AFM tip changes from 0 to 10 μm, the force increases from 0 to 39.6 nN (using the AFM’s force calibration) resulting in a deflection of the U-shape tapered fiber. As shown in Fig. 2B, the output signal from the U-shape STMS fiber structure was also periodic with a frequency of 1 Hz and was synchronized and in phase with the AFM output signal. It can be concluded that the U-shape STMS fiber structure has an ultrahigh force sensitivity which has the potential to replace the AFM assuming a suitable calibration is carried out. However, the noise level for the detected signal from the InGaAs-APD (using a gain setting of 40 dB) is high; therefore, to reduce the noise level, high frequencies (>25 Hz) are removed by low-pass filtering. A linear fit is also applied to the data points in the rising and falling edges of the filtered triangular signal waveform, as shown in Fig. 2C. The force signal applied by the piezo actuator to the U-shape STMS fiber structure varies from 0 to 39.6 nN, which results in peak-to-peak amplitude of 10.75 mV for the detected signal in Fig. 2C. The conversion factor for the detected voltage versus applied force is therefore 3.68 nN/mV (calculated from 39.6 nN /10.75 mV). The noise only of the experimental system is shown in Fig. 2D, which is obtained when no force is applied to the U-shape fiber, the variation in the voltage is ±0.49 mV. Assuming the limit of detection (LoD) is defined as three times the maximum noise voltage (3 × 0.49 = 1.47 mV), then using the conversion factor of 3.68 nN/mV, the estimated LoD of the developed U-shape STMS fiber sensor is 1.47 mV × 3.68 nN/mV ≈ 5.4 nN.

The U-shape STMS fiber structure can measure force from the deflection of the U-shape tapered fiber, assuming a suitable calibration takes place between fiber deflection and applied force. Since the AFM can measure force accurately, it can be used to provide such a calibration. Figure 2E shows the relationship between the fiber displacement and the applied force for the three different U-shape STMS fiber sensors, which confirms good linearity for all the sensors. The slope of the force versus displacement characteristic increases for larger taper waist diameters, this is expected; since as the diameter of the taper increases, the taper becomes mechanically stiffer and the taper movement for a given applied force reduces. The spring constants of the three U-shape STMS fiber structures are shown in Table 1.

To verify the repeatability of force measurement, during calibration, the force measurements for each fiber sensor were repeated four times. Figure 2F shows the average measured forces for the three U-shape STMS fiber structures (sensors 1 to 3) with average values of 39.6, 61.7, and 79.6 nN and an SD of 0.2, 1.1, and 2.7 nN, respectively. These results demonstrate that the sensors show good repeatability with relatively small error bars, which, in turn, reinforces the real-world potential of the structure as a viable sensor to detect weak force levels.

### Demonstration application: The measurement of the spring constant of a human hair

**Fig. 3.**
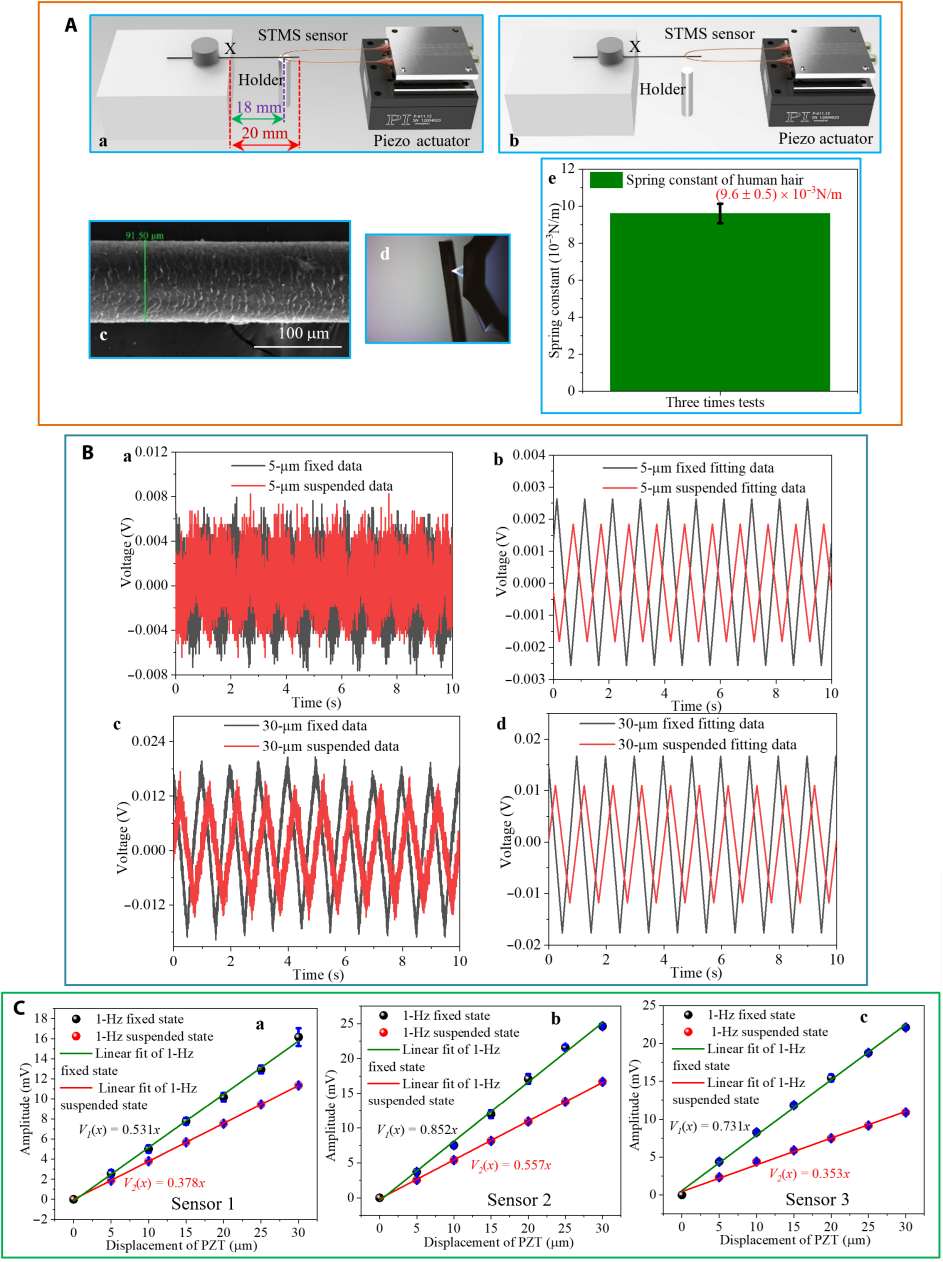
Human hairspring constant measurements. (**A**) Schematic diagram of the STMS sensor for human hair force detection for the (a) hair in contact with the metal post, (b) hair-suspended state, (c) SEM image of a human hair (female adult), (d) microscope image of AFM tip in contact with the human hair, and (e) AFM measured spring constant of human hair. (**B**) Response comparison of the 5-μm fixed and suspended states: (a) raw data and (b) fitted data; response comparison of the 30-μm fixed and suspended states: (c) raw data and (d) fitted data. (**C**) The relationship between amplitude and displacement of the piezo actuator for the 1-Hz fixed and suspended states with error bars for the three sensor samples: (a) sensor 1, (b) sensor 2, and (c) sensor 3.

To demonstrate practical force measurement using the proposed U-shape microfiber interferometer, the three fabricated U-shape STMS fiber sensor samples described above were used to measure the spring constant of a human hair (female adult). The length of the suspended section of the hair is 20 mm (Fig. 3A-a). SEM images have been taken to measure the diameter of the human hair used in the experiments and the results show that the diameter varies from 81 to 92 μm along the length of the sample hair. Figure 3A-c shows an example of an SEM image over a short length of hair, where the measured hair diameter is 91.5 μm.

Figure 3A shows a schematic diagram of the experimental setup for hair force measurement. As shown in Fig. 3A, one end of the sample human hair is fixed at point X to a translation stage, and the other end is suspended in air. By carefully adjusting the translation stage upward, the suspended end of the human hair can be brought into contact with the center of the U-shape STMS fiber structure.

A piezo actuator (P-611.ZS, PI), shown in Fig. 3A, is used to control the movement of the STMS fiber structure, and it is also attached to a translation stage to allow vertical adjustment. The piezo actuator operates at a frequency of 1 Hz with a movement amplitude range of 5 to 30 μm. The experiment involved two distinct steps:

Step (I): A metal post is placed underneath the suspended human hair to prevent it from deflecting when an external force is applied to the hair, where the distance from the hair support point is 18 mm (Fig. 3A-a). The U-shape STMS fiber is precisely displaced vertically downward so that it makes contact with the human hair, at a point on the hair 18 mm away from the fixed-point X. In this case, since the human hair is supported by the post, a displacement of the U-shape STMS fiber by the piezo actuator is converted into a deflection of the U-shape STMS fiber. This step allows for the relationship between the displacement of the U-shape STMS fiber and the detected signal amplitude to be determined, providing a calibration of the U-shape STMS fiber sensor. Six different displacement amplitudes of 5, 10, 15, 20, 25, and 30 μm were applied to the U-shape STMS fiber and the detected signals were recorded.

Step (II): The holder (Fig. 3A-b) post is removed so that the hair is unsupported. The vertical position of the U-shape STMS fiber tip is adjusted so that it maintains contact with the hair, at a point 18 mm from the fixed point of the hair. In this case, a displacement of the U-shape STMS fiber by the piezo actuator results in a deflection of both the hair and the U-shape STMS fiber. As in step (I), six different displacement amplitudes of 5, 10, 15, 20, 25, and 30 μm were applied to the U-shape STMS fiber. In this case, the ratio between the slopes in the hair-suspended state (without the holder) and the hair-fixed state (with the holder) can be used in the calculations of the spring constant of the hair (see Eqs. 1 to 5).

Figure 3B shows an example of the recorded signal changes of the U-shape STMS fiber (sensor 1) for step (I) (hair-fixed state) and for step (II) (hair-suspended state) for displacements of 5 and 30 μm at a frequency of 1 Hz. For both displacements, the raw detected signals were filtered and fitted as described previously to produce a processed signal for further analysis. It can be seen that the signal amplitudes for the hair-suspended state are lower than those for the hair-fixed state. This is because the reaction force applied to the U-shape STMS fiber by the hair is lower than the reaction force applied by the hair when it is supported by the metal post in the hair-fixed state. In effect, in the hair-suspended state, the hair deflects when the U-shape STMS fiber tip makes contact with the hair, reducing the net deflection of the U-shape STMS fiber and thus reducing the amplitude of the detected signal from the sensor.

The relationship between the peak signal voltage amplitude and the applied displacement is shown in Fig. 3C for both the hair-fixed state and hair-suspended state for each of the three sensors. In addition, the measurements were repeated three times for each sensor; error bars are shown in each plot in Fig. 3C to reflect this. On the basis of a linear fit, the relationships between the displacement and the measured voltages *V_f_* and *V_s_* for the fixed state and suspended state respectively are the following

$$\text{Hair}-\text{fixed state}:V_{f}=a\;x$$ (1)

$$\text{Hair}-\text{suspended state}:V_{s}=b\;x$$ (2)

The ratio *R* of the slopes of the two linear fits is *b/a*, which can be interpreted as the factor by which the detected voltage is reduced, for the hair-suspended state compared to the hair-fixed state, for the same values of the displacement *x*. Since the detected voltage amplitude from the U-shape STMS fiber is linearly proportional to the force applied to the fiber tip, therefore the relationship between the reaction forces applied to the U-shape STMS fiber for the two states is also given by *R*. The values of the ratio *R* for the three U-shape STMS fiber sensors (sensors 1 to 3) are 0.71, 0.65, and 0.48 respectively.

In the hair-suspended state, with the U-shape STMS fiber tip in contact with the hair, both the hair and the U-shape STMS fiber can be treated as a pair of simple springs coupled together, forming a series arrangement of springs. For convenience, the two springs are referred to hereinafter as the “hairspring” and the “fiber spring.” When a displacement is applied to the U-shape STMS fiber by the piezo actuator, both the hairspring and the fiber spring will deflect. As the two springs form a series spring arrangement, the sum of the two deflections is equal to the value of the displacement.

As a pair of springs arranged in series, the equivalent spring constant *k*_eq_ of the two springs together can be found from the following formula (*32*)

$$\frac{1}{k_{\text{eq}}}=\frac{1}{k_{h}}+\frac{1}{k_{f}}$$ (3)

where *k_h_* is the unknown spring constant of the hair and *k_f_* is the spring constant of the fiber tip. The values of *k_f_* for the three U-shape STMS fiber sensors are already known from the AFM calibration described earlier in this work and are shown in Table 1. The value of *k*_eq_ can be found as follows. The total deflection of the two springs together is equal to the applied displacement of *D* μm. In the suspended hair state, when a total displacement of *D* μm is applied to the U-shape STMS fiber, the force can be calculated as *k_f_DR* which is the product of the force in the fixed hair state *k_f_D* nN and the ratio *R* calculated above. From Hooke’s law, the spring constant is the ratio of the applied force and the displacement, thus the value of *k*_eq_ can be calculated as

$$k_{\text{eq}}=\frac{k_{f}\mathrm{DR}}{D}=k_{f}R$$ (4)

thus we have

$$k_{h}=\frac{R}{1-R}k_{f}$$ (5)

For the three U-shape STMS fiber sensors (sensors 1 to 3), the value of the spring constant of the hair sample can be calculated using Eq. 5. The results are shown in Table 2. To verify this result, the spring constant of the same hair was measured using the AFM alone. The measurement was repeated three times and an average was taken. Figure 3A-d shows a microscope image of the contact between the AFM tip and the hair. The average spring constant of the hair measured using AFM alone is 9.60 × 10^−3^ N/m (Fig. 3A-e), which is close to the measurements by our proposed U-shape STMS fiber sensors, shown in Table 2, with the AFM measured value shown for comparison.

**Table 2.** Measured spring constant using the STMS fiber sensor.

Sensor	Sensor 1	Sensor 2	Sensor 3	Average AFM measured value
Spring constant	9.79 × 10^−3^ N/m	9.75 × 10^−3^ N/m	10.92 × 10^−3^ N/m	9.60 × 10^−3^ N/m

To determine the maximum force this sensor can detect, a 60-μm displacement with a step change of 5 μm was applied to sensor 3. The value of 60 μm was set by the upper working limit of the available piezo actuator. The result shows that the amplitude of the sensor signal increases linearly as displacement increases. Since the force calibrated by AFM shows that a 6.73-μm displacement results from a 79.6-nN force (Fig. 2F), the maximum force measured in this experiment is 709.6 nN. Note that the measurement of larger forces could be achieved by allowing for a larger displacement to be applied to the fiber sensor. Alternatively, a fiber sensor with a larger taper waist diameter could be used.

Table 3 compares the performance of several different optical fiber force sensors, with mention of the sensor structure, force sensitivity, detection limit, and the approximate physical size of the sensor. From Table 3, it is clear that the force sensitivity of the proposed U-shape microfiber interferometer presented in this work is much higher than that of other sensors.

**Table 3.** Performance comparisons of different optical fiber force sensors. FBG, Fiber Bragg grating.

Sensor structure	Detected minimum force	Approximate sensor size	References
Balloon-like interferometer	235.2 μ**N**	24 mm × 14 mm	(*27*) (2018)
Clamped beam probe	300 n**N**	68 μm × 100 μm	(*30*) (2021)
Microfiber asymmetrical FP interferometer	−33.1 μ**N**	20 mm × 7.3 μm	(*34*) (2014)
FBG fabricated in uniform-waist fiber tapers	2 m**N**	8 mm × 19 μm	(*35*) (2011)
Surface-corrugated microfiber Bragg grating	2.07 m**N**	112 μm × 2.5 μm	(*36*) (2012)
FP micro-cavity plugged by cantilever taper	0.049 **N**	1360 μm × 125 μm	(*37*) (2021)
FP cuboid cavity	49 m**N**	18 μm × 60 μm	(*38*) (2021)
Photonic crystal fiber	0.098 **N**	125 μm × 3 cm	(*39*) (2020)
Microfiber Bragg grating	1.37 m**N**	2.4 mm × 2.4 mm	(*40*) (2012)
**U-shape probe (this work)**	**39.6 nN (sensor 1)**	**3 mm × 30 mm**	**Proposed method in this paper**

## DISCUSSION

Here, an ultrahigh sensitivity all-fiber force sensor based on a configuration of a U-shape STMS fiber structure is proposed and experimentally investigated. The LoD for force is estimated to be as low as ~5.4 nN. The proposed U-shape microfiber interferometer has many advantages, such as a simple fabrication technique, high mechanical strength, an ultralow detection limit, and is physically compact. In future work, we will investigate the use of the proposed sensor in the measurement of piconewton-level interaction forces in the field of mechanobiology, including cellular and molecular mechanics.

## MATERIALS AND METHODS

### Fabrication of the U-shape microfiber interferometer

**Fig. 4.**
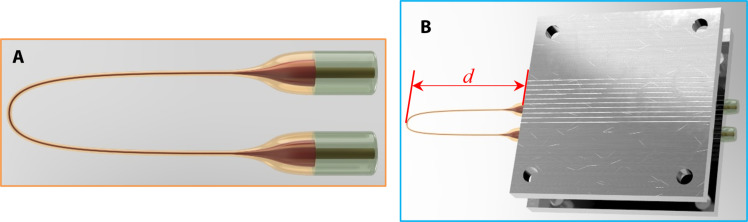
U-shape microfiber sensor structure. (**A**) Schematic diagram of U-shape tapered microfiber interferometer. (**B**) Schematic diagram of U-shape microfiber package.

Figure 4 shows a schematic diagram of the U-shape tapered microfiber interferometer, which is based on an STMS fiber structure. A multimode fiber (MMF; 105/125, FG105LCA, Thorlabs, 10-mm length) is spliced between two SMFs (Type SMF28) by a traditional fusion splicer (Fujikura 70C). The MMF was tapered to form a microfiber using a tapering setup based on a ceramic heater and two motorized translation stages. The two SMFs connected to the MMF are fixed on the two translation stages. Under the control of a PC, the translation stages move the fiber structure repeatedly through the ceramic heater which operates at about 1000°C, progressively tapering the MMF section to a waist diameter in the range of 4 to 6 μm. The MMF section is then carefully bent to form a U-shape STMS fiber structure as shown in Fig. 4A.

The fabricated U-shape STMS is then fixed on a fiber holder (a custom-designed metal plate with inscribed V-grooves), where the length of the suspend section *d* is 30 mm (as shown in Fig. 4B). The input light propagates from the SMF to the MMF, travels through the U-shape microfiber section, and then to the output SMF. Because of the multimode interference occurring within the tapered MMF, the STMS fiber structure is very sensitive to any changes to the U-shape (*33*). Any small force applied to the U-shape microfiber induces minor deformation of the microfiber, leading to a change in the output power from the STMS fiber structure. Since the microfiber has a very small diameter (4 to 6 μm), the spring constant of the U-shape microfiber is very small, and thus, very small levels of force can introduce detectable deformation of the U-shape microfiber. Assuming that calibration is carried out between the output power and the force applied to the U-shape microfiber, the STMS fiber structure can measure contact forces in the order of nanonewtons.
