# Supplementary material for: A nanonewton force sensor using a U-shape tapered microfiber interferometer
Source: Sci Adv. 2024 May 29;10(22):eadk8357. doi: 10.1126/sciadv.adk8357 (PMC11135392; doi:10.1126/sciadv.adk8357)
Supplement: Supplementary file 1 — Legend for movie S1 [file sciadv.adk8357_sm.pdf]

Science Advances
AAAS

Supplementary Materials for

A nanonewton force sensor using a U-shape tapered microfiber interferometer

Ling Chen *et al.*

Corresponding author: Jinhui Yuan, yuanjinhui81@bupt.edu.cn; Qiang Wu, qiang.wu@northumbria.ac.uk

Sci. Adv. **10**, eadk8357 (2024)
DOI: 10.1126/sciadv.adk8357

The PDF file includes:

Legend for movie S1

Other Supplementary Material for this manuscript includes the following:

Movie S1

Movie S1: Measured signal responses from the AFM and from U-shape STMS fiber structures with a bend diameter of 3 mm, in which a periodic triangular movement waveform (10 μm peak-peak in amplitude at a frequency of 1 Hz) is applied to the U-shape STMS by a piezo actuator in the AFM.
